# Age matters in orthodontics: divergent motivations, perfectionism, and patient-clinician discord in treatment need assessment

**DOI:** 10.3389/fmed.2025.1714237

**Published:** 2026-01-21

**Authors:** Guo Zhao, Fengjie Zhu

**Affiliations:** Department of Stomatology, The First Affiliated Hospital of Zhengzhou University, Zhengzhou, China

**Keywords:** orthodontics, pediatrics, perfectionism, psychology, surveys and questionnaires

## Abstract

**Background:**

Understanding age-related differences in the psychological aspects of orthodontic treatment is crucial for patient-centered care. This study compared treatment motivation, psychosocial impact, and perfectionism between child and adult patients.

**Methods:**

In this cross-sectional study, 306 orthodontic patients (223 children, 83 adults) completed validated questionnaires assessing motivation, psychosocial impact (PIDAQ), and perfectionism (FMPS). Patient-perceived aesthetic need (IOTN-AC) and orthodontist-evaluated clinical need (IOTN-DHC) were recorded.

**Results:**

Adults reported significantly higher total treatment motivation (*p* = 0.021) and a markedly greater psychosocial impact of malocclusion (PIDAQ: 58 vs. 35, *p* < 0.001) than children, with higher scores in social, psychological, and aesthetic concern domains. Adults also showed stronger functional motivation (*p* = 0.004), greater concern over mistakes (*p* = 0.018), and lower parental expectations (*p* = 0.029). A significant discrepancy existed between patient self-perceived need (IOTN-AC) and orthodontist assessment (IOTN-DHC) in both groups (*p* < 0.001). Regression analysis showed that higher psychosocial impact (PIDAQ) was associated with female gender, older age, higher perfectionism, and greater self-perceived aesthetic need, but not with clinical need (IOTN-DHC). The total PIDAQ score was the only significant independent predictor of higher treatment motivation (β = 0.10, *p* = 0.002).

**Conclusion:**

Adult orthodontic patients experience a greater psychosocial burden and distinct motivational drivers compared to children. Treatment motivation is primarily driven by psychosocial distress, not clinical severity, highlighting the need for clinicians to prioritize patient-reported outcomes and address perception gaps to deliver effective, patient-centered care.

## Introduction

As orthodontics transitions from traditional biomedical paradigms toward biopsychosocial models, it has become essential for clinicians to understand patients’ psychological states. Malocclusion significantly affects oral health–related quality of life (OHRQoL), influencing oral function, orofacial appearance, and psychosocial wellbeing, as widely documented in the literature (1). However, most studies rely on generic oral health assessment instruments such as OHIP-14, which fail to adequately capture patient perceptions of dental aesthetics or orthodontics-specific quality of life (2). To address this gap, the Psychosocial Impact of Dental Aesthetics Questionnaire (PIDAQ) was developed as a condition-specific OHRQoL instrument to evaluate patients’ sensitivity to social interactions, negative emotions, and satisfaction with dental appearance, thereby reflecting the perceived psychosocial impact of tooth aesthetics (3).

In clinical research, orthodontic treatment need is commonly assessed using instruments such as the Index of Orthodontic Treatment Need (IOTN), which incorporates both anatomical and aesthetic perspectives. The IOTN consists of two components: the Dental Health Component (IOTN-DHC), based on clinical occlusal characteristics, and the Aesthetic Component (IOTN-AC), which reflects the patient’s self-perceived need for treatment based on aesthetic criteria (4). Understanding the interplay between these measures and their correlation with treatment outcomes is vital for comprehensive patient-centered care.

Motivation for orthodontic treatment is influenced by a variety of factors, including the desire to improve dental appearance and the psychosocial effects of malocclusion (5). Such motivations may differ considerably across age groups, as children and adults exhibit distinct expectations and concerns shaped by their developmental stages. Although these distinctions are well-recognized, comparative studies examining age-related differences in psychological profiles, including perfectionism and psychosocial impact, remain limited. Moreover, the extent to which patient-clinician discord in treatment need assessment varies by age has not been thoroughly investigated.

Personality traits, particularly perfectionism, also play a significant role in moderating psychosocial responses to dental aesthetics and motivation for treatment. While traits such as self-esteem, depression, and obsessive-compulsiveness have been examined among orthodontic patients (6, 7), perfectionism has received limited attention in orthodontic populations, especially in relation to age differences. The Frost Multidimensional Perfectionism Scale (FMPS) (8) offers a reliable tool for assessing perfectionism in orthodontic settings. Incorporating these personality dimensions can enhance our understanding of their influence on treatment motivation and OHRQoL. To integrate these diverse factors into a coherent framework, researchers have turned to conceptual models such as the Wilson and Cleary health-related quality of life (HRQoL) model (9). This model bridges biomedical and psychosocial aspects of health, providing a structured approach to examining the relationships among clinical variables, individual characteristics, health perceptions, and overall HRQoL.

Therefore, this study aimed to fill these gaps by comparing treatment motivation, psychological status, and perfectionism between child and adult orthodontic patients, with age, treatment need (IOTN-DHC/IOTN-AC), and FMPS as exposure variables, and motivation and PIDAQ as outcome variables. Secondary aims included examining relationships among these variables and assessing patient-orthodontist agreement in treatment need evaluation. The null hypotheses posited no age-based differences in motivation, psychosocial impact, or perfectionism; no interrelationships among HRQOL factors; and no discrepancy in treatment need assessments between patients and orthodontists.

## Materials and methods

### Study design and participants

This cross-sectional study was approved by the Ethics Committee of The First Affiliated Hospital of Zhengzhou University and conducted in accordance with the STROBE guidelines. A convenience sampling method was used to recruit patients seeking orthodontic treatment at the hospital between January 2023 and December 2024.

Sample selection and criteria involved screening all new patients presenting for an initial orthodontic consultation for eligibility, where inclusion required individuals to be either under 18 years of age (classified as the child/adolescent group) or adults (≥18 years) with no history of prior orthodontic treatment. A total of 350 patients were approached during the study period. Of these, 306 agreed to participate and completed the questionnaires, yielding a response rate of 87.4%. To assess potential non-response bias, we compared the age and gender distribution of the participants with that of the 44 non-respondents using available clinic records. No significant differences were found in age (*p* = 0.320) or gender distribution (*p* = 0.410), suggesting that the study sample was representative of the patient population seeking treatment at the clinic. The exclusion criteria encompassed the presence of cognitive impairment or communication barriers preventing comprehension of the questionnaires, a diagnosis of chronic systemic diseases that could influence oral health or treatment perception, congenital craniofacial anomalies or noticeable facial scars, or severe temporomandibular disorders or active, severe periodontal disease. To minimize selection bias, no restrictions were applied regarding gender or type of malocclusion, and written informed consent was obtained from all adult participants and from the guardians of minors, with additional verbal assent provided by child participants.

### Data collection and outcome measures

Following the initial clinical examination, eligible participants completed a demographic form and a set of validated questionnaires. The primary outcome measures were treatment motivation and the psychosocial impact of malocclusion. The secondary outcome measures were perfectionism and the level of agreement between patient and orthodontist assessments of treatment need.

The IOTN-AC was used to assess the patient’s self-perceived aesthetic need. Patients selected the photograph from a 10-grade scale that most closely matched their own dental appearance. For analysis, grades 1–4 were categorized as mild/no need, grades 5–7 as moderate need, and grades 8–10 as severe need.

The IOTN-DHC was used by the orthodontist to assess the clinical need for treatment based on dental casts. It evaluates occlusal traits such as overjet, overbite, and crossbite. Grades 1–2 were categorized as mild/no need, grade 3 as moderate need, and grades 4–5 as severe need.

The 12-item Treatment Motivation Questionnaire was used to assess motivation across three domains: energizing (general willingness to undergo treatment), esthetics (desire to improve appearance), and functioning (desire to improve oral function). This instrument was adapted from Meade and Inglehart (10). The Chinese version was developed through a standardized process of forward- and back-translation, and its psychometric properties, including construct validity and internal consistency, were confirmed in a prior study of Chinese orthodontic patients (8). Items are rated on a five-point Likert scale (1 = strongly disagree to 5 = strongly agree), with higher total and domain scores indicating stronger motivation.

This 23-item PIDAQ measures the psychosocial impact of malocclusion across four domains: dental self-confidence (reverse-scored), social impact, psychological impact, and aesthetic concern. Responses are given on a five-point Likert scale (0 = not at all to 4 = very much). Higher scores indicate a greater negative psychosocial impact, except for the dental self-confidence subscale, where a higher score indicates greater confidence.

The Chinese version of the FMPS (8) assesses perfectionism across five subscales: concern over mistakes, personal standards, parental expectations, doubts about actions, and organization. Items are rated on a five-point Likert scale (1 = strongly disagree to 5 = strongly agree). Higher scores reflect stronger perfectionist traits.

Concurrently, a single trained and calibrated orthodontist, who was blinded to the patients’ questionnaire responses, assessed the malocclusion severity using the IOTN-DHC based on dental casts. Intra-examiner reliability was confirmed by re-evaluating 30 randomly selected cases after a one-month interval, yielding an excellent intraclass correlation coefficient (ICC) of 0.85.

### Statistical analysis

All statistical analyses were performed using R software (version 3.4.4, R Foundation for Statistical Computing, Vienna, Austria). Descriptive statistics were presented as frequencies and percentages for categorical variables. For continuous variables, the data distribution was assessed for normality using the Shapiro-Wilk test. As the data significantly deviated from a normal distribution (*p* < 0.05 for most variables), non-parametric tests were employed for inferential analysis.

The Mann-Whitney U test was used to compare continuous variables (e.g., motivation scores, PIDAQ scores, FMPS scores) between children and adults. The McNemar’s test was applied to evaluate the differences in the categorical classification of orthodontic treatment need (IOTN-AC vs. IOTN-DHC) between patients and the orthodontist. The Spearman’s rank correlation coefficients (ρ) were calculated to determine the strength and direction of associations between total and domain scores of the various measures (e.g., motivation, PIDAQ, FMPS, IOTN) within each age group. Multiple linear regression analyses were conducted to identify factors associated with the total PIDAQ score and high treatment motivation. The assumptions of linear regression (linearity, homoscedasticity, independence of errors, and normality of residuals) were checked using residual plots and diagnostic tests. The variance inflation factor (VIF) was used to check for multicollinearity, with all VIF values below 2, indicating no significant multicollinearity. In the models, gender was coded as 1 for male and 2 for female.

The threshold for statistical significance was set at a two-tailed *p*-value ≤ 0.05. A *p*-value < 0.01 was considered highly significant.

## Results

### Baseline data

In total, 306 patients were enrolled, stratified by age group (children: *n* = 223; adults: *n* = 83). The sample was slightly female-predominant overall (158 females, 148 males), with a higher proportion of males among children (123 males vs. 100 females) and a markedly higher proportion of females among adults (58 females vs. 25 males). Assessment of self-perceived orthodontic treatment need using the Index of IOTN-AC revealed that the majority of patients reported mild or no need (*n* = 206), while 38 reported moderate need and 62 reported severe need. A similar pattern was observed with the IOTN-DHC, where 81 patients reported mild/no need, 81 moderate need, and 144 severe need based on self-perception. Notably, children constituted the majority in all IOTN-AC and IOTN-DHC severity categories, reflecting their larger representation in the overall sample. These baseline data provide a foundation for comparing perceived treatment need, psychosocial impact, and motivation between pediatric and adult populations in the context of orthodontic care (Table 1).

**TABLE 1:** Baseline data of the enrolled patients.

Variable	Total (*n* = 306)	Children (*n* = 223)	Adult (*n* = 83)
**Gender**			
Male	148	123	25
Female	158	100	58
**On the basis of self-perceived IOTN-AC**			
Mild/no (1–4)	206	157	49
Moderate (5–7)	38	22	16
Severe (8–10)	62	44	18
**On the basis of self-perceived IOTN-DHC**			
Mild/no (1–2)	81	63	18
Moderate (3)	81	62	19
Severe (4–5)	144	98	46

### Subjective psychological and motivational data

Significant differences were observed in overall treatment motivation and specific domains of psychosocial impact and perfectionism. Adults reported significantly higher total motivation for treatment compared to children (52 vs. 47, *p* = 0.021), with the most notable difference in the functioning index (21 vs. 19, *p* = 0.004), suggesting a stronger drive among adults to improve oral function. While no significant differences were found in the energizing or esthetics indices, the psychosocial impact of malocclusion was markedly greater in adults, as evidenced by significantly higher total PIDAQ scores (58 vs. 35, *p* < 0.001), indicating a more pronounced negative psychosocial experience. This was driven by significant differences in the social impact (SI), psychological impact (PI), and aesthetic concern (AC) subscales, all of which were higher in adults (*p* < 0.001). Dental self-confidence (DSC) did not differ significantly between groups. Regarding perfectionism, although total FMPS scores were similar, adults showed significantly higher concern over mistakes (CM) (11 vs. 9, *p* = 0.018) and lower personal standards in the esthetics-related domain (PE) (14 vs. 15, *p* = 0.029), suggesting a more critical self-evaluation in adult patients. These findings highlight age-related differences in the psychological and motivational profiles of orthodontic patients, with adults experiencing greater psychosocial burden and functional motivation despite similar overall perfectionism levels (Table 2).

**TABLE 2:** Subjective data and differences between children and adult groups.

Variable	Children	Adults	*P*
**Motivation for treatment**			
Total motivation	47 (40–56)	52 (44–58)	0.021
Energizing index	15 (13–20)	16 (15–20)	0.232
Esthetics index	11 (8–16)	12 (10–15)	0.124
Functioning index	19 (16–24)	21 (18–24)	0.004
**Psychosocial impact**			
Total PIDAQ	35 (25–54)	58 (40–68)	<0.001
DSC (PIDAQ)	22 (17–25)	23 (16–25)	0.887
SI (PIDAQ)	5 (1–15)	15 (5–23)	<0.001
PI (PIDAQ)	8 (4–14)	16 (9–20)	<0.001
AC (PIDAQ)	1 (0–5)	7 (2–10)	<0.001
**Perfectionism**			
Total FMPS	75 (67–88)	81 (65–91)	0.318
CM (FMPS)	9 (5–15)	11 (7–19)	0.018
PS (FMPS)	18 (14–23)	19 (15–22)	0.575
PE (FMPS)	15 (12–20)	14 (9–15)	0.029
DA (FMPS)	11 (8–14)	12 (10–15)	0.475
OR (FMPS)	75 (67–89)	80 (70–90)	0.222

### Agreement between patient self-perceived and orthodontist-evaluated treatment need

The results reveal a statistically significant discrepancy between patient and clinician assessments in both age groups (*p* < 0.001). Among children, a substantial proportion of those who perceived their malocclusion as mild or having no treatment need (*n* = 157) were classified by orthodontists as having moderate (*n* = 45) or severe (*n* = 62) need according to IOTN-DHC. Similarly, among adults, many who self-rated as having mild/no need (*n* = 49) were clinically assessed as having moderate (*n* = 17) or severe (*n* = 18) treatment need. Furthermore, a notable number of patients in both groups who perceived moderate or even severe aesthetic need were also categorized into various DHC levels, indicating inconsistent alignment between subjective perception and clinical judgment. This significant mismatch underscores the discordance between how patients—both children and adults—perceive the severity of their dental aesthetics and how orthodontists evaluate functional and health-related aspects of malocclusion. The findings highlight the importance of integrating both patient-reported outcomes and clinical assessments in treatment planning to address both psychosocial concerns and objective dental health needs (Table 3).

**TABLE 3:** Difference of the Index of Orthodontic Treatment Need (IOTN) evaluated by Aesthetic Component (AC) and Dental Health Component (DHC) of children and adults.

IOTN-AC	IOTN-DHC			*P*
Children	Mild/no (*n* = 63)	Moderate (*n* = 62)	Severe (*n* = 98)	
Mild/no (*n* = 157)	50	45	62 18	
Moderate (*n* = 22)	2	2		
Severe (*n* = 44)	11	15	18	<0.001
**Adults**	**Mild/no** **(*n* = 18)**	**Moderate** **(*n* = 19)**	**Severe** **(*n* = 46)**	
Mild/no (*n* = 49)	14	17	18 13	
Moderate (*n* = 16)	2	1		
Severe (*n* = 18)	2	1	15	<0.001

### Factors associated with PIDAQ score and high treatment motivation

For total PIDAQ, female gender (Beta coefficients = 1.86, *p* = 0.017), older age (Beta coefficients = 1.35 per year, *p* < 0.001), higher perfectionism (total FMPS; Beta coefficients = 1.28 per point, *p* < 0.001), and greater self-perceived aesthetic need (IOTN-AC; Beta coefficients = 1.60 per grade, *p* < 0.001) were significantly associated with increased psychosocial impact, indicating that these factors independently contribute to a more negative perception of dental aesthetics. In contrast, clinical treatment need (IOTN-DHC) was not significantly associated with PIDAQ scores (*p* = 0.198). When analyzing predictors of high treatment motivation, only the total PIDAQ score emerged as a significant independent predictor (Beta coefficients = 1.10 per point, *p* = 0.002), suggesting that patients who experience greater psychosocial distress due to their dental appearance are more motivated to seek orthodontic treatment. Gender, age, perfectionism, and both IOTN-AC and IOTN-DHC did not show statistically significant associations with motivation in the multivariable model. These findings highlight that while psychosocial and perceptual factors strongly influence emotional wellbeing related to dental aesthetics, it is primarily this psychosocial burden—not clinical need or personality traits—that drives treatment motivation in orthodontic patients (Table 4).

**TABLE 4:** Multivariable regression analysis with total Psychosocial Impact of Dental Aesthetics Questionnaire (PIDAQ) or motivation as the dependent variable.

Independent variable	Variable	β [95% CI]	*P*
PIDAQ	Gender (female vs. male)	1.86 [1.02–2.98]	0.017
	Age (per year increase)	1.35 [1.13–1.66]	<0.001
	Total FMPS (per point increase)	1.28 [1.11–1.53]	<0.001
	IOTN-AC (per grade increase)	1.60 [1.19–2.08]	<0.001
	IOTN-DHC (per grade increase)	1.16 [0.79–1.64]	0.198
High treatment motivation	Gender (female vs. male)	1.00 [0.57–1.65]	0.967
	Age (per year increase)	1.06 [0.91–1.20]	0.876
	Total PIDAQ (per point increase)	1.10 [1.05–1.22]	0.002
	Total FMPS (per point increase)	1.10 [0.92–1.47]	0.325
	IOTN-AC (per grade increase)	0.96 [0.86–1.28]	0.486
	IOTN-DHC (per grade increase)	0.81 [0.60–1.18]	0.135

## Discussion

Understanding patients’ psychological states from their subjective perspective is crucial for orthodontists in the pre-treatment phase. Grounded in the Wilson and Cleary health model, our study provides insights into the associations between age, psychological factors, and orthodontic perceptions. The results of this cross-sectional study suggest malocclusion can exert a significant negative impact on patients’ self-concept and social interactions, reinforcing the value of a patient-centered approach that goes beyond clinical metrics. The first null hypothesis was partially rejected, as significant differences were observed between children and adults across multiple dimensions of motivation, psychosocial impact, and perfectionism. Similarly, the second null hypothesis was also partially rejected, given the significant associations found between exposure variables and outcomes.

A growing body of literature consistently demonstrates that both adolescents and adults primarily seek orthodontic treatment to improve dental aesthetics (11). Our findings align with this trend, revealing that both age groups placed high value on aesthetic outcomes, as reflected in similar scores on the esthetics index of the motivation questionnaire. However, a key distinction emerged: adults exhibited significantly higher motivation to improve oral function. This observation is consistent with previous research indicating that functional concerns become more salient with age. For instance, Samsonyanová and Broukal’s systematic review identified facial attractiveness as the primary motivational factor, particularly among younger patients (5). In contrast, Pabari et al. (12) reported that adult patients are motivated by both aesthetic enhancement and functional improvements. Our findings extend this understanding by directly comparing children and adults, demonstrating that while aesthetic motivation remains strong across age groups, functional motivation increases significantly in adulthood. This shift may be attributed to greater life experience and the realization that malocclusion can impact speech clarity, chewing efficiency, and long-term dental health.

Moreover, the observed age-related differences in parental influence are noteworthy. Adults reported significantly lower scores on the parental expectations domain of the perfectionism scale, indicating that they perceive less pressure from parents regarding their orthodontic treatment. This finding is consistent with developmental theories suggesting that autonomy increases with age (13) and is supported by Wedrychowska-Szulc and Syryńska (14), who found that parental influence on the decision to undergo orthodontic treatment diminishes with age. In contrast, children are more likely to internalize parental standards. Clinically, this underscores the importance of tailoring communication strategies: with children, it is essential to involve parents and align expectations, whereas with adult patients, the focus should be on individual goals and self-perceived needs.

Interestingly, adults also exhibited higher levels of concern over mistakes, a dimension of perfectionism that reflects anxiety about making errors or falling short of standards. This suggests that adult patients may experience greater psychological distress related to perceived dental imperfections. Tung and Kiyak (15) previously noted that both children and parents anticipate significant improvements in self-esteem following orthodontic treatment, but our findings indicate that adults may have higher internalized standards and are more self-critical. This heightened sensitivity to imperfection could lead to increased anxiety during treatment and higher expectations for ideal outcomes. Clinically, this implies that adult patients may require more psychological support and realistic goal-setting to manage treatment-related stress and prevent dissatisfaction, even with objectively successful results. However, the interpretation of these perfectionism scores warrants caution regarding cultural specificity. The FMPS, while widely used and validated in its Chinese version, was developed within a Western cultural framework. Concepts such as “Parental Expectations” and “Concern over Mistakes” may manifest and be weighted differently in collectivist societies like China, where familial harmony and social evaluation can hold distinct significance. For instance, high scores on “Parental Expectations” in children might reflect a culturally normative family dynamic rather than pathological pressure. Therefore, while our findings highlight meaningful age-related trends, a deeper, qualitative exploration or the development of a culturally-grounded instrument might be necessary to fully capture the nuances of perfectionism in this patient population and to validate whether the FMPS subscales carry equivalent conceptual meaning across the age groups studied.

The discrepancy between IOTN-AC and IOTN-DHC is another critical finding. A substantial proportion of patients who perceived their malocclusion as mild or non-existent were classified by orthodontists as having moderate or severe treatment needs based on dental health criteria. This mismatch highlights a perceptual gap between patients and clinicians. While clinicians focus on occlusal function and long-term dental health, patients are often primarily concerned with aesthetics. This divergence is not unique to our study; Feu et al. (16) found that adolescents with more severe aesthetic impairments reported poorer OHRQoL and were more likely to seek treatment. Our results reinforce the need for effective communication to bridge this gap, helping patients understand the functional and health implications of malocclusion.

The strongest predictor of high treatment motivation in our multivariable model was the total PIDAQ score, indicating that psychosocial burden is a key driver for seeking treatment. This finding supports a “psychosocial drive” model of orthodontic motivation, where emotional distress related to dental appearance acts as a primary catalyst. This is consistent with the concept of “possible selves” introduced by Meade and Inglehart (10) and applied to adolescents by Anderson et al. (17). Our data suggest that this concept is also relevant for adult populations, showing that the desire to alleviate current psychosocial distress is a powerful motivator. However, it is important to note that while PIDAQ was a significant predictor, the regression model explained only a portion of the variance in treatment motivation, indicating that other unmeasured factors (e.g., cost, social support, access to care) also play important roles.

Furthermore, our regression analysis revealed that female gender, older age, higher perfectionism, and greater self-perceived aesthetic need were independently associated with higher psychosocial impact, while clinical need was not. This reinforces the idea that subjective perception, rather than objective clinical severity, is a stronger determinant of psychological wellbeing in this context. This aligns with a longitudinal study by Nichols et al. (18), which found that changes in self-perceived dental aesthetics were associated with psychosocial wellbeing in adulthood.

This study has several limitations that should be considered when interpreting the results. First, the cross-sectional design precludes any causal inferences about the relationships between psychological factors, motivation, and treatment-seeking behavior. Second, the use of convenience sampling from a single university hospital may limit the generalizability of the findings to other cultural or clinical settings. Third, while we controlled for several variables, residual confounding from unmeasured factors (e.g., socioeconomic status, previous dental experiences) is possible. Furthermore, although the Chinese versions of the questionnaires have been validated, subtle cultural interpretations of certain items or concepts may still exist and could influence responses. The self-reported nature of these instruments also introduces the possibility of recall and social desirability biases, where participants might provide answers they perceive as more socially acceptable. Additionally, the IOTN-AC, being a visual scale, relies on patient self-perception which can be subjective and influenced by factors beyond dental aesthetics alone, such as overall facial appearance and self-esteem (19, 20). Finally, the difference in sample size between the child and adult groups, though reflective of typical clinic demographics, may have affected the statistical power for some subgroup analyses.

In conclusion, this study demonstrates that adult orthodontic patients report a greater psychosocial burden from malocclusion and are more motivated by functional concerns than children. Crucially, treatment motivation was primarily driven by this psychosocial distress rather than the clinical severity of the malocclusion, and a significant perception gap existed between patient and clinician assessments of treatment need. These findings underscore the importance of incorporating patient-reported psychosocial outcomes into orthodontic practice to complement clinical evaluations. However, given the cross-sectional nature of this single-center study, the identified associations require validation through longitudinal research in more diverse populations. Future studies should also investigate the causal pathways linking psychological factors to treatment-seeking behavior. Ultimately, adopting a biopsychosocial approach that addresses both the physical and emotional dimensions of malocclusion is essential for delivering truly patient-centered care.

## Data Availability

The original contributions presented in this study are included in this article/supplementary material, further inquiries can be directed to the corresponding author.
